# Pelvic floor disorders and impact on sexual function: a cross-sectional study among non–sexually active and sexually active women

**DOI:** 10.1093/sexmed/qfae024

**Published:** 2024-05-09

**Authors:** Signe Nilssen Stafne, Silje Kristine Sveen Ulven, Tone Prøsch-Bilden, Susan Saga

**Affiliations:** Clinic of Rehabilitation, St Olavs Hospital, Trondheim University Hospital, 7006 Trondheim, Norway; Department of Public Health and Nursing, Norwegian University of Science and Technology, 7491 Trondheim, Norway; Faculty of Medicine and Health Sciences, Norwegian University of Science and Technology, 7491 Trondheim, Norway; Norwegian National Advisory Unit on Incontinence and Pelvic Floor Health, University Hospital of North Norway, 9019 Tromsø, Norway; Department of Public Health and Nursing, Norwegian University of Science and Technology, 7491 Trondheim, Norway

**Keywords:** sexual function, sexual dysfunction, PISQ-IR, pelvic floor disorders, menopause, pelvic organ prolapse, urinary incontinence, anal incontinence, colorectal-anal distress, PFDI-20

## Abstract

**Background:**

Pelvic floor disorders are common and associated with impaired sexual function in women.

**Aim:**

To assess women with pelvic floor disorders and describe factors associated with not being sexually active and those associated with sexual function in sexually active women.

**Methods:**

A cross-sectional study was conducted that included nonpregnant women with symptoms of pelvic floor disorders who were referred to the urogynecologic and surgical outpatient clinic at 2 Norwegian university hospitals: St Olavs Hospital, Trondheim University Hospital, and the University Hospital of Northern Norway, Tromsø. Women answered a questionnaire anonymously.

**Outcomes:**

Pelvic Organ Prolapse Incontinence Sexual Questionnaire–IUGA Revised.

**Results:**

Of 157 respondents, 111 (71%) reported being sexually active (with or without a partner), and 46 (29%) reported not being sexually active. As compared with sexually active women, not sexually active women were older (mean ± SD, 60.2 ± 13.3 vs 51 ± 12.1 years; *P* < .001), more were menopausal (78% vs 47%, *P* = .001), and more had symptom debut <1 year (31% vs 9%, *P* < .001). They reported more distress related to pelvic floor disorders, especially pelvic organ prolapse. In a multivariate logistic regression analysis, menopausal women and women with symptom debut <1 year were 4 times more likely to be not sexually active than premenopausal women (odds ratio, 4.0; 95% CI, 1.7-9.2) and women with symptom debut ≥1 year (odds ratio, 4.0; 95% CI, 1.5-10.7). In sexually active women, colorectal-anal distress was negatively associated with 5 of 6 domains of sexual function: arousal/orgasm (ß = –0.36; 95% CI, –0.02 to –0.005), partner related (ß = –0.28; 95% CI, –0.01 to –0.002), condition specific (ß = –0.39; 95% CI, –0.002 to –0.009), global quality (ß = –0.23; 95% CI, –0.02 to –0.002), and condition impact (ß = –0.34; 95% CI, –0.02 to –0.006).

**Clinical Implications:**

Health care professionals should discuss sexual function in patients with pelvic floor disorders, especially menopausal women and women with colorectal-anal symptoms.

**Strengths and Limitations:**

The study used condition-specific measures and recruited women from 2 university hospitals with wide range of age. Limitations include the small sample size and wide confidence intervals. The number of women who considered themselves not sexually active was low, and item nonresponse levels among these women where somewhat high. Of 625 eligible women, 200 (32%) answered the questionnaire. Sexual health and sexual function are still surrounded with taboo, and some women were probably not comfortable answering the questions.

**Conclusion:**

Menopausal women and women with recent onset of symptoms of pelvic floor disorders are more likely to be sexually inactive, and colorectal-anal symptoms have the most negative impact on sexual function in sexually active women.

## Introduction

Pelvic floor disorders (PFDs) are common in women, with 25% reporting urinary incontinence (UI)^1^ and 19% anal incontinence (AI)^2^ and with anatomic pelvic organ prolapse (POP) present in up to 50% of parous women, of which 20% report symptoms.^3^ UI, AI, and POP are often coexisting, with unique and shared risk factors.^4^ The etiology of PFD is complex, with pregnancy and delivery as unique and established risk factors in younger women and with menopause and general aging in older women.^5^

Experiencing PFD is associated with declined psychological, social, physical, and sexual well-being. Female sexual function is complex, consisting of physiologic, anatomic, psychological, and social-interpersonal components. Sexual functioning is defined as “absence of difficulty moving through the stages of sexual desire, arousal, and orgasm, as well as subjective satisfaction with the frequency and outcome of individual and partnered sexual behavior.”^6^ One review study found the prevalence of sexual dysfunction to be 30% to 50% in the general female population. In women with PFD, the prevalence was increased to 50% to 83%.^7^

Although the World Health Organization considers sexual health to be fundamental to the overall health and well-being of individuals,^8^ there is limited knowledge on how women with PFD perceive their sexual function. A qualitative study in women with POP found that they changed sexual intimacy practices or totally avoided sexual intercourse because of embarrassment or discomfort.^9^ Other studies have reported poorer sexual function, reduced frequency or sexual inactivity, anxiety, depression, shame, embarrassment, and fear of soiling or worsening the symptoms.^7^^,^^10^^,^^11^ The aim in the present study is to further assess sexual function in women with PFDs. Our research questions were as follows: Among women with PFDs, (1) which factors are associated with not being sexually active and (2) which factors are associated with sexual function in sexually active women with PFD?

## Methods

### Participants and study design

This is a secondary analysis of data collected during validation of the Norwegian translation of the Pelvic Organ Prolapse Incontinence Sexual Questionnaire–IUGA Revised (PISQ-IR).^12^ The recruitment period was June 2020 to June 2021. Participants eligible for study inclusion were women aged >18 years who were nonpregnant and able to read Norwegian; who were experiencing symptoms of POP, AI, or UI; and who were referred to the urogynecologic or surgical outpatient clinic at St Olavs Hospital, Trondheim University Hospital, and the University Hospital of Northern Norway, Tromsø. We excluded women with painful bladder syndrome, vulvodynia, or other chronic pelvic pain for >6 months. Eligible women were sent an invitation to participate and the questionnaire via a prepaid envelope with the letter of appointment for the first hospital visit.

### Outcome variables

The primary outcome was sexual function assessed with the PISQ-IR, which was developed in 2013 by the International Urogynecological Association and is a condition-specific validated assessment instrument.^12^ The revised version is based on a multicultural framework and provides a reliable instrument for use in many cultures. The PISQ-IR has been translated and validated in the Norwegian female population (manuscript under production) through several steps: (1) translation of the questionnaire from English to Norwegian by a bilingual translator; (2) testing for readability, comprehensibility, and equivalence through cognitive interviews with 10 women with PFDs; (3) assessment by multidisciplinary clinical PFD experts; (4) another 10 cognitive interviews with women with PFDs; (5) translation of the questionnaire back into English by an independent bilingual translator; and (6) review and approval of this version of the questionnaire by the IUGA Translation Working Group. Discrepancies were identified and amended between steps 2 and 4.

The respondents defined themselves as not sexually active (NSA) or sexually active (SA), alone or with a partner, initially in the PISQ-IR. NSA is divided into 4 domain-specific subscales and SA into 6 domain-specific subscales^13^ (Table 1). The PISQ-IR domain-specific subscale scores were calculated with a transformed sum method.^13^ Each domain gives a score between 0 and 100. In NSA women, a higher score indicates poorer sexual function, whereas for SA women, a higher score indicates better sexual function.

**Table 1:** Subscales of the Pelvic Organ Prolapse Incontinence Sexual Questionnaire–IUGA Revised.^13^

**NSA: not sexually active**		**SA: sexually active**	
**Domain**	**Related questions**	**Domain**	**Related questions**
*NSA-CS*: condition-specific reasons for not being active	*Q2c*: Due to bladder or bowel problems (urinary or fecal incontinence) or due to prolapse (a feeling of or a bulge in vaginal area) *Q2d*: Because of my other health problems *Q2e*: Pain	*SA-CS*: assessment of condition-specific impacts on activity	*Q8b*: Shame *Q8c*: Fear *Q9*: How often do you leak urine and/or stool with *any type* of sexual activity?
*NSA-PR*: partner-related reasons for not being active	*Q2a*: No partner *Q2b*: No interest	*SA-PR*: assessment of partner-related impacts	*Q13*: How often does your partner have a problem (lack of arousal, desire, erection etc) that limits your sexual activity? *Q14a*: Your sexual desire *Q14b*: The frequency of your sexual activity
*NSA-GQ*: global quality rating of sexual quality	*Q4a*: Satisfied to dissatisfied *Q4b*: Adequate to inadequate *Q5a*: I feel frustrated by my sex life *Q6*: Overall, how bothersome is it to you that you are not sexually active?	*SA-GQ*: global quality rating of sexual quality	*Q19a*: Satisfied to dissatisfied *Q19b*: Adequate to inadequate *Q19c*: Confident to not confident *Q20a*: I feel frustrated by my sex life
*NSA-CI*: condition impact on sexual quality	*Q3*: How much does *fear* of leaking urine and/or stool and/or a bulging in the vagina (either the bladder, rectum or uterus falling out) cause you to *avoid or restrict* your sexual activity? *Q5b*: I feel sexually inferior because of my incontinence and/or prolapse *Q5c*: I feel angry because of the impact that incontinence and/or prolapse has on my sex life	*SA-CI*: condition-specific impact on sexual quality	*Q18*: How much does the fear of leaking urine, stool and/or a bulging in the vagina (prolapse) cause you to avoid sexual activity? *Q20b*: I feel sexually inferior because of my incontinence and/or prolapse *Q20c*: I feel embarrassed about my sex life *Q20d*: I feel angry because of the impact that incontinence and/or prolapse has on my sex life
		*SA-AO*: assessment of arousal, orgasm	*Q7*: How often do you feel sexually aroused (physically exited or turned on) during sexual activity? *Q8a*: Fulfilled *Q10*: Compared with orgasms you have had in the past, how intense are your orgasm now? *Q11*: How often do you feel pain during sexual intercourse?
		*SA-D*: assessment of sexual desire	*Q15*: When you are involved in sexual activity, how often do you feel that you want more? *Q16*: How frequently do you have sexual desire, this may include wanting to have sex, having sexual thoughts or fantasies, etc? *Q17*: How would you rate your level (degree) of sexual desire or interest?

Explanatory variables were demographic data and symptoms and severity of PFDs collected with self-reported questionnaires. Demographic data were age, body mass index, parity, menopausal status, previous surgery for PFDs, and previous or ongoing conservative treatment for PFDs. To assess UI, we used the International Consultation on Incontinence Questionnaire–UI Short Form (ICIQ-UI SF): an outcome measure developed to assess prevalence, severity, impact on quality of life, and situations of urinary leakage.^14^ The score ranges from 0 to 21, with a higher score indicating more severe symptoms. The St Marks incontinence score was used to assess the frequency of loose and solid stool leakage, gas leakage, impact on daily life, urgency, pad use, and use of constipating medication.^15^ The score ranges from 0 to 24 with higher scores indicating more severe symptoms. The Pelvic Floor Distress Inventory Questionnaire–20 (PFDI-20)^16^ consists of 3 scales: Colorectal Anal Distress Inventory–8 (CRADI-8), Pelvic Organ Prolapse Distress Inventory–6 (POPDI-6), and Urinary Distress Inventory–6 (UDI-6). Each scale gives a score ranging from 0 to 100, and the PFDI-20 total score ranges from 0 to 300. The higher the score, the more severe the distress.^16^^,^^17^

### Statistics

Statistical analyses were performed with SPSS Statistics for Windows (version 27.0; IBM Corp). Descriptive statistics for categorical variables are presented as frequencies and percentages, while continuous variables are presented with mean, SD, and range. A chi-square test was performed to compare differences among categorical variables and an independent-samples *t*-test to compare continuous variables.

Factors associated with being NSA were explored by multivariable logistic regression analysis, and multivariable linear regression was used to explore factors associated with being SA. We included variables from univariable analyses with *P* < .20 in the multivariable analysis. None of the variables in the multivariable regression models were highly correlated (variance inflation factor <2.0). Effect estimates are presented as odds ratio (OR) and coefficients (ß) with 95% CIs. *P* < .05 was considered statistically significant.

*R* ^2^ was used to assess how well the predictors in the chosen model explained the dependent variable. Normally distributed residuals were found in variables included in the multivariable linear regression through a visual assessment of Q-Q plots,^18^^,^^19^ and correlations between the dependent factor and the explanatory factors were linear.^20^ As recommended, no replacements were made for missing data.^13^

### Ethical considerations

The study was approved by the Regional Committee for Medical and Health Research Ethics (95426) and the institutional review boards at the Department of Obstetrics and Gynecology and the Department of Surgery at the 2 hospitals (St Olavs Hospital, Trondheim, and the University Hospital of Northern Norway, Tromsø). Permission to translate the PISQ-IR was granted by the International Urogynecological Association. All data were collected anonymously, and descriptive data are compared on a group level.

## Results

**Figure 1:**
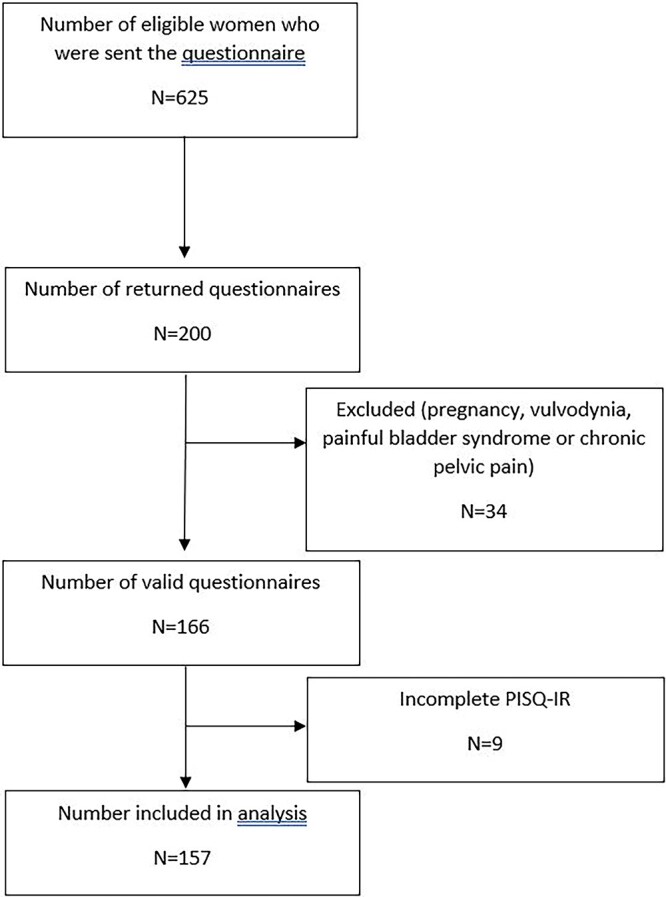
Flowchart of invited, responding, and included women.

The study invitation and questionnaire were sent to 625 patients, and 200 returned the questionnaire (response rate, 32%). Of these, 34 were excluded due to exclusion criteria, and 9 women were additionally excluded due to missing data on the PISQ-IR. In total, 157 women were included in the present analysis (Figure 1).

In the study population, 46 (29%) reported being NSA and 111 (71%) being SA (with or without a partner). NSA women were older and more were menopausal. Furthermore, NSA women had a shorter duration since PFD symptom debut as compared with SA women (Table 2). The presence and severity of AI and UI and the levels of urinary distress and colorectal-anal distress were comparable between NSA and SA women. NSA women had a higher level of POP distress and higher total score on the PFDI-20. Domain-specific subscale scores of the PISQ-IR in SA and NSA women are presented in Table 3.

**Table 2:** Characteristics of study population (N = 157).^a^

	**Not sexually active (n = 46)**	**Sexually active (n = 111)**	***P* value**
Age, y	60.2 ± 13.3 (27-78)	51.3 ± 12.1 (25-76)	<.001
Missing	1	4	
Body mass index, kg/m^2^	27.5 ± 5.8 (13.8-42.6)	25.9 ± 4.3 (14.0-41.8)	.067
Missing	1	3	
Menopause	36 (78)	52 (47)	<.001
Missing		2	
Previous or ongoing local/systemic estrogen treatment	20 (61)	33 (67)	.449
Missing	4	3	
Parity			.834
0	3 (7)	8 (7)	
1	7 (15)	13 (12)	
≥2	36 (78)	90 (81)	
PFD symptom debut			.001
<6 mo	5 (11)	3 (3)	
6-12 mo	9 (20)	7 (6)	
1-5 y	19 (41)	47 (42)	
6-10 y	7 (15)	22 (20)	
>10 y	6 (13)	30 (27)	
Missing		2	
PFD symptom debut <1 y	14 (31)	10 (9)	<.001
Missing		2	
Previous surgery for PFD	17 (37)	26 (23)	.076
Missing	2	4	
Previous/ongoing conservative treatment for PFD	31 (67)	88 (79)	.107
Missing	2	4	
Other diseases^b^	16 (35)	31 (28)	.383
Missing	1	2	
Previously sought professional help for sexual dysfunction^c^	0/26	2/109	—
Missing	20	2	
Pelvic floor symptoms			
Urinary incontinence	38 (84)	90 (81)	.620
Missing	1		
ICIQ-UI SF^d^	10.6 ± 4.6 (3-20)	9.9 ± 4.7 (2-21)	.434
Anal incontinence	34 (79)	88 (82)	.734
Missing	3	3	
St Marks index^e^	8.1 ± 5.0 (1-19)	7.1 ± 6.1 (1-20)	.057
PFDI-20	115.5 ± 57.1 (33.3-242.7)^f^	96.0 ± 49.8 (10-256.3)	.037
POPDI-6	37.8 ± 26.0 (0-91.7)	27.5 ± 21.5 (0-87.5)	.011
CRADI-8	32.1 ± 25.2 (0-81.3)^g^	31.1 ± 24.2 (0-93.8)	.816
UDI-6	44.3 ± 24.6 (0-95.8)^g^	37.5 ± 23.9 (0-100)	.115

Abbreviations: CRADI-8, Colorectal Anal Distress Inventory–8; ICIQ-UI SF, International Consultation on Incontinence Questionnaire–UI Short Form; PFD, pelvic floor disorder; PFDI-20, Pelvic Floor Distress Inventory Questionnaire–20; POPDI-6, Pelvic Organ Prolapse Distress Inventory–6; UDI-6, Urinary Distress Inventory–6.

a Data are presented as mean ± SD (range) or No. (%).

b Diseases that can lead to urinary incontinence, anal incontinence, and pelvic organ prolapse, such as diabetes, neurologic disease, depression, use of antidepressants, and previous radiation therapy of the pelvis.

c The number of patients who have sought help/the total number of patients who have answered the question.

d Calculated for women reporting ICIQ-UI SF ≥1.

e Calculated for women reporting St Marks ≥1.

f n = 44.

g n = 45.

**Table 3:** Subscales of PISQ-IR scoring reported as transformed score (range, 0-100).

	**Mean ± SD (95% CI); No. (missing %)**	
	**Not sexually active (n = 46)** ^a^	**Sexually active (n = 111)** ^b^
CS: condition specific	50 ± 35 (37-63); 30 (35)	77 ± 25 (72-81); 100 (10)
PR: partner related	69 ± 33 (59-80); 38 (17)	76 ± 21 (72-81); 99 (11)
GQ: global quality	52 ± 33 (42-63); 41 (11)	52 ± 28 (47-57); 110 (1)
CI: condition impact	46 ± 40 (33-58); 40 (13)	67 ± 33 (60-73); 110 (1)
AO: assessment of arousal/orgasm	—	63 ± 19 (59-67); 111 (0)
D: desire	—	47 ± 18 (44-51); 111 (0)

a Higher score indicates a higher impact on sexual function.

b Higher score indicates better sexual function.

In NSA women, menopausal status, shorter symptom debut, and POPDI-6 showed a significance level <.2 in the univariable logistic regression analyses and were thus included in the multivariable analysis. In the multivariable logistic regression analyses, we found menopausal status (OR, 4.0; 95% CI, 1.7-9.2) and symptom debut <1 year (OR, 4.0; 95% CI, 1.5-10.7) as factors significantly associated with being NSA (Table 4).

**Table 4:** Multivariable logistic regression analysis: factors associated with not being sexually active.

	**Univariable**		**Multivariable**	
	**OR (95% CI)**	***P* value**	**aOR (95% CI)**	***P* value**
PFD symptom debut <1 y	4.3 (1.8-10.7)	.001	4.0 (1.5-10.7)	.005
Menopausal	3.9 (1.8-8.7)	<.001	4.0 (1.7-9.2)	.001
POPDI-6	1.02 (1.00-1.03)	.013	1.02 (1.0-1.03)	.058

Abbreviations: aOR, adjusted odds ratio; OR, odds ratio; PFD, pelvic floor disorder; POPDI-6, Pelvic Organ Prolapse Distress Inventory–6.

When we assessed factors associated with being SA, the following showed a significance level <.2 in the univariable regression analyses and were thus included in the multivariable analysis: age, body mass index, duration of PFD symptoms, previous PFD surgery, previous or ongoing conservative treatment, other diseases (depression, use of antidepressants, neurologic disease, diabetes, and previous radiation therapy of the pelvis), POPDI-6, CRADI-8, and UDI-6. Table 5 shows that colorectal-anal distress demonstrated a significantly negative association with SA–arousal/orgasm (ß = –0.36; 95% CI, –0.02 to –0.005), SA–partner related (ß = –0.28; 95% CI, –0.01 to –0.002), SA–condition specific (ß = –0.39; 95% CI, –0.002 to –0.009), SA–global quality (ß = –0.23; 95% CI, –0.02 to –0.002), and SA–condition impact (ß = –0.34; 95% CI, –0.02 to –0.006). Having other diseases was negatively associated with SA–global quality (ß = –0.24; 95% CI, –0.98 to –0.12); urinary distress was negatively associated with SA–condition specific (ß = –0.22; 95% CI, –0.02 to –0.001); and higher age was negatively associated with SA–arousal/orgasm (ß = –0.22; 95% CI, –0.026 to –0.003). Furthermore, previous PFD surgery was positively associated with SA–desire (ß = 0.21; 95% CI, 0.03-0.70), and having POP distress was negatively associated with SA–condition impact (ß = –0.19; 95% CI, –0.02 to –0.000).

**Table 5:** Multivariable linear regression models: domain-specific subscales and factors associated with being sexually active.

	**Model: effect variable, ß (95% CI); *P* value**					
	**1: SA-AO (n = 102)**	**2: SA-PR (n = 93)**	**3: SA-CS (n = 90)**	**4: SA-GQ (n = 105)**	**5: SA-CI (n = 100)**	**6: SA-D (n = 102)**
Definition	Arousal/orgasm	Partner related	Condition specific	Global quality	Condition impact	Desire
**Predictor**						
Age	–0.22 (–0.026, –0.003); .018	–0.14 (–0.02, 0.003); .16	0.12 (–0.006, 0.03); .20	–0.1 (–0.02, 0.008); .31	0.13 (–0.004, 0.03); .15	–0.15 (–0.02, 0.003); .09
Body mass index			–0.09 (–0.06, 0.02); .32			
Duration of PFD			–0.18 (–0.34, –0.001); .048			
Previous PFD surgery	–0.08 (–0.48, 0.19); .38	–0.12 (–0.47, 0.11); .23			–0.13 (–0.71, 0.14); .18	0.21 (0.03, 0.70); .03
Conservative PFD treatment^a^			–0.12 (–0.78, 0.16); .20		–0.18 (–0.93, 0.014); .06	–0.19 (–0.73, 0.01); .06
Other diseases^b^	–0.07 (–0.44, 0.22); .50		–0.15 (–0.73, 0.06); .09	–0.24 (–0.98, –0.12); .01	–0.05 (–0.53, 0.29); .57	
POPDI-6	–0.04 (–0.008, 0.005); .66		–0.01 (–0.008, 0.009); .91		–0.19 (–0.02, 0.000); .053	
CRADI-8	–0.36 (–0.02, –0.005); <.001	–0.28 (–0.01, –0.002); .007	–0.39 (–0.02, –0.009); <.001	–0.23 (–0.02, –0.002); .02	–0.34 (–0.02, –0.006); <.001	–0.16 (–0.01, 0.001); .11
UDI-6	–0.10 (–0.01, 0.003); .32		–0.22 (–0.02, –0.001); .03		–0.09 (–0.01, 0.004); .35	–0.05 (–0.007, 0.004); .62
**Overall statistics**						
*R* ^2^	0.25	0.12	0.41	0.13	0.29	0.13
*F* (*df*), *P* value	5.171 (6, 95); <.001	4.043 (3, 89); .01	7.126 (8, 81); <.001	4.967 (3, 101); .003	5.367 (7, 92); <.001	2.812 (5, 96); .02

Abbreviations: CRADI-8, Colorectal Anal Distress Inventory–8; PFD, pelvic floor disorder; POPDI-6, Pelvic Organ Prolapse Distress Inventory–6; SA, sexually active; UDI-6, Urinary Distress Inventory–6.

a Previous/ongoing treatment for PFD.

b Diseases that can lead to urinary incontinence, anal incontinence, and pelvic organ prolapse, such as diabetes, neurologic disease, depression, use of antidepressants, and previous radiation therapy of the pelvis.

## Discussion

We found that NSA women were older and more were menopausal; they also had a shorter duration of PFD symptom debut and higher pelvic floor distress, especially related to POP. After adjusting for potential confounders, menopausal women and women with PFD symptom debut <1 year were >4 times likely to be NSA as compared with premenopausal women and women with PFD symptom debut ≥1 year. In SA women, colorectal-anal distress was negatively associated with 5 of 6 domains of sexual function.

Age is a significant predictor of declined sexual activity and function,^21^^,^^22^ with a strong association between menopausal status and low sexual function.^23^ The menopausal transition is a time characterized by hormonal, physiologic, and social changes. Also with aging, there is an inevitable decline in health and increasing use of medications. Morbidity and poor self-assessed general health are associated with decreased sexual function,^23^^,^^24^ after adjusting for age.^23^

One interesting finding is that NSA women had a shorter time since onset of PFD symptoms as compared with SA women. No difference was found in occurrence of PFD symptoms and severity of UI and AI, although NSA women reported more distress related to PFD and POP. A qualitative study including women with symptomatic POP revealed that many completely avoid physical intimacy and sexual intercourse due to shame and embarrassment associated with the condition.^9^ Furthermore, fear of worsening the prolapse has been identified as 1 factor among women who reduce sexual activity following POP.^10^ It might be that women with new symptoms adjust their sexual priorities and practices to cope with physiologic and bodily changes because they are not familiar with their symptoms yet.

Having a partner is one of the most important predictors of women’s sexual activity and sexual satisfaction.^21^^,^^25^ Among our NSA women, the partner-related domain had the highest score, indicating the highest impact on sexual function. A decline in women’s sexual activity and function may also be a result of the partner’s age, health, and sexual function.^22^ Our results are comparable to other European and Asian studies assessing female sexual function with the PISQ-IR such that NSA women were older^26–33^ and the partner-related domain had the highest impact on sexual function.^27^^,^^28^^,^^30^^,^^31^^,^^33^

In our population, 71% of the participating women defined themselves as SA (alone or with a partner). In multivariable analysis, we found significant negative associations between higher colorectal-anal distress and 5 of the 6 domain-specific subscales. Colorectal-anal distress was measured with the CRADI-8, which is a symptom inventory to measure the degree of bother and distress caused by multiple colorectal-anal symptoms, including AI, urgency, emptying difficulties, and pain during defecation. Previous literature is mainly limited to AI and sexual function. Pauls et al noted that women with AI had similar rates of sexual activity as women without AI but poorer sexual function as measured with the PISQ-IR.^11^ Similarly, Imhoff et al stated that most women with AI are SA but report more sexual dysfunction.^34^ Fear of soiling during intercourse and embarrassment are factors for reduced sexual function in women with AI.^7^ Urinary distress was negatively associated with the condition-specific subdomain, while distress related to POP was negatively associated with the condition impact subdomain. Coital or orgasmic incontinence is of significant concern for women with UI, while worries about the image of their vagina, embarrassment, concerns about a partner’s satisfaction, reduced sensation, and discomfort are reported in women with POP.^7^

In SA women, the subdomains with the lowest score and thus the highest impact on sexual function were desire and global quality. The highest impact of desire is noted in other studies assessing female sexual function with the PISQ-IR.^27^^,^^30^^,^^31^^,^^33^

There is strong evidence that PFD has a negative impact on female sexual function and that the coexistence of PFDs has a cumulative negative effect on sexual function.^7^^,^^35^ Sexual function is complex and varies across populations and throughout the life course. The Natsal-3 survey cited an overall prevalence of 51.2% women reporting at least 1 sexual function problem lasting ≥3 months in the past year. The proportion of SA women reporting 1 or more problems increased steadily with age.^23^ Another survey indicated that 74.2% reported sexual activity in the past year and only 50.8% reported sexual satisfaction.^25^ A recent study with normative data of sexual domains in Norwegian women found a decrease in sexual functioning, sexual enjoyment, libido, sexual satisfaction, and importance of sexual activity by age.^24^ Furthermore, symptoms of vaginal dryness and worry of incontinence increased with age.^24^ Despite the high prevalence of female sexual dysfunction, sexuality is a neglected topic in health care.

The average age of menopause is 52 years, and women are expected to live 25 to 30 years after menopause. Since sexual health is such an important part of quality of life, women should be given the opportunity to talk about their sexual problems as a fundamental part of health care.^36^ In a recent Norwegian study, women had a very low score on satisfaction with communication with professionals despite generally low sexual function.^24^ Older women had the lowest sexual function and lowest satisfaction in communication with professionals.^24^ Sexuality is still taboo, and patients may be embarrassed to request help or are unaware about treatment options. It is essential to educate health care professionals how to talk about sexual function. Furthermore, there is an urgent need for research on the effects of conservative, medical, and surgical treatment of PFD on sexual function.^7^ Importantly, condition-specific measures of sexual function should be included in such studies.

This study contributes to a field with scant knowledge and in need for increased focus. The strength of this study is the use of a condition-specific measure, recruitment from 2 university hospitals, and anonymously collected data. Furthermore, the study included women in a wide age range, which improves external validity. However, the study has some limitations. Since this is a secondary analysis, no a priori power calculation was performed. The overall sample size was small, and confidence intervals were wide. The number of women who considered themselves NSA were especially low. We have no knowledge about women not responding to the study. Sexual health and sexual function are still topics with taboo. It might be that some invited women were not comfortable answering the questions. In line with Rockwood et al,^13^ item nonresponse levels in NSA were somewhat high and missing in the 4 subdomains, varying between 11% and 35%. Assessing sexual function in women who do not consider themselves sexually active is challenging.

## Conclusion

Age, menopause, and shorter duration of PFD symptoms are associated with being NSA. The partner-related domain had the most impact on sexual function in NSA women. For SA women, colorectal-anal distress was most negatively associated with sexual function. Desire and global quality were the subdomains with the highest impact on sexual function. Sexual function is complex and multifactorial. However, health care workers must be confident in the topic and address sexual health during consultation in women with PFDs.

## Notes

The paper is derived from a thesis at: https://ntnuopen.ntnu.no/ntnu-xmlui/handle/11250/2993297
